# Relationship Between Sexual Dysfunction and Quality of Life in Psychiatric Patients Receiving Regular Treatment: A Cross-Sectional Study

**DOI:** 10.7759/cureus.67116

**Published:** 2024-08-18

**Authors:** Siva Prasad Damam, Veerabadram Yeshala, Ramasubba Reddy Mukkara, Vivaswan Boorla, Rakesh Kotha

**Affiliations:** 1 Department of Psychiatry, Osmania Medical College, Hyderabad, IND; 2 Department of Psychiatry, Government Medical College, Vikarabadh, Vikarabadh, IND; 3 Department of Neonatology, Osmania Medical College, Hyderabad, IND

**Keywords:** councelling, schizophrenia, antipsychotics, quality-of-life, sexual dysfunction, psychiatry & mental health

## Abstract

Background: Sexual dysfunction (SD) is a significant issue among psychiatric patients on psychotropic medications. This study aims to compare SD in patients using antipsychotics and antidepressants.

Objective: To evaluate the prevalence of SD and its effect on the quality of life among psychiatric patients on medications.

Methods: A cross-sectional study had been conducted with 150 participants (50 with schizophrenia, 50 with affective disorders, and 50 controls). SD was evaluated employing the Psychotropic-Related Sexual Dysfunction Questionnaire (PRSexDQ) and the Arizona Sexual Experience Scale (ASEX). The Quality of Life Enjoyment and Satisfaction Questionnaire-Short Form (Q-LES-Q-SF) was used to measure the quality of life. Data analysis techniques included Spearman's correlation test, one-way ANOVA, chi-square test, and descriptive statistics.

Results: SD prevalence was 42% in affective disorders, 64% in schizophrenia, and 18% in controls. SD significantly correlated with a longer duration of psychotropic use and negatively impacted quality of life.

Conclusion: SD is prevalent among psychiatric patients on long-term medication, highlighting the need for strategies to manage these side effects.

## Introduction

Human sexuality encompasses an individual's sexual interest, attraction, and capacity for erotic experiences and responses based on sexual orientation. Over time, the understanding of sexuality has evolved significantly, moving beyond procreation to include aspects that enhance the quality of life (QoL) and well-being [1]. The notion of what is considered "normal" in sex varies widely among individuals and across cultures. Despite the growing body of knowledge on the topic, myths about sexual functioning persist, particularly those pertaining to the effect of psychiatric medications on sexual function. Contrary to popular belief, the effects of certain psychiatric medications on sexual functioning are minimal [2].

Sexual dysfunction (SD) is the inability to engage in sexual activity for enjoyment, interest, or physiological reasons [3]. Reduced sexual desire, lubrication deficiencies, erectile dysfunction, and difficulty experiencing an orgasm are some of the symptoms of SD. These issues can affect QoL, relationships, and self-esteem. Epidemiological studies carried out in India have shown that 25-25% of the general population and 25-95% of those on psychotropic medications have SD [4]. One's QoL is greatly harmed by these dysfunctions, which can lead to non-compliance with treatment and unfavorable attitudes toward therapy [5].

Sexual health is often neglected in clinical practice, especially in psychiatric settings, despite being a critical component of overall health and well-being. The stigma associated with sexual health issues and mental health disorders exacerbates the situation by causing underreporting and undertreatment of SD. SD must be addressed if treatment outcomes, sexual health, and overall QoL are to be improved for psychiatric patients. Moreover, the physiological and psychological interaction between sexual function and mental health disorders necessitates a comprehensive approach to patient care. The ability to procreate can be negatively impacted by medications used to treat mental health conditions, particularly antipsychotics and antidepressants [6]. Addressing these interconnected issues requires a holistic approach to treatment that considers both mental and sexual health as integral components of overall well-being.

The reluctance to discuss sexual issues openly in many cultures, including in India, can lead to further complications. Patients might be hesitant to report their symptoms due to embarrassment or fear of judgment, resulting in a lack of diagnosis and treatment. Hence, it is essential for healthcare providers to initiate conversations about sexual health proactively and create a supportive environment where patients feel comfortable discussing their concerns.

## Materials and methods

Participants and research design

The Institute of Mental Health (IMH) in Erragadda, Hyderabad, Telangana, conducted this cross-sectional, comparative study. The study involved 150 participants divided into three groups: 50 with schizophrenia, 50 with affective disorders, and 50 controls. Participants were selected based on inclusion criteria: ages 18-50, diagnosed with schizophrenia or affective disorders according to the International Classification of Diseases (ICD-10), on regular medication for the past six months, and in remission with a regular sexual partner. Exclusion criteria included other psychiatric illnesses, chronic co-morbid medical conditions, intellectual disability, or substance dependence. Epi Info software (Centers for Disease Control and Prevention, Atlanta, GA) was used to compute the sample size. The study was conducted from January 2023 to January 2024 over a period of one year.

Procedure

Prior to commencing the study, approval (approval no.: ECR/300/AP/2023/RR-16) was issued by the institutional ethics committee, Osmania Medical College, Hyderabad. The study, which took place at the IMH, Hyderabad, focused on patients undergoing regular treatment, medication, and follow-up for schizophrenia and affective disorders. In addition to this, 50 members of the general public without any history of psychiatric illness were included in the study to assess SD and QoL in these groups. Following a detailed explanation of the method and obtaining signed informed consent, participants who met the inclusion and exclusion criteria were recruited. Participants were informed of their right to withdraw from the study at any time and that their participation was entirely voluntary. A 26-page semi-structured questionnaire capturing sociodemographic data and information on the participants' psychotropic medication and duration of medication use was administered. The guidelines of the ICD-10 were applied to diagnose schizophrenia and affective disorders, and the modified Kupuswam scale was used to assess socioeconomic status.

Data collection

A semi-structured pro forma was used to collect data, and on it were details about psychotropic medications, their duration of use, and sociodemographic data. The Psychotropic-Related Sexual Dysfunction Questionnaire (PRSexDQ) was employed to assess SD, and the Arizona Sexual Experience Scale (ASEX) was utilized to evaluate sexual function. The Quality of Life Enjoyment and Satisfaction Questionnaire-Short Form (Q-LES-Q-SF) was employed to evaluate QoL [7,8]. 

Statistical analysis

Descriptive statistics were employed to characterize the research population. Means as well as standard deviations were employed to analyze categorical data, and frequencies and percentages were utilized to analyze continuous variables. For continuous variables, the Spearman's correlation test was used; for categorical data correlations, the chi-square test and one-way ANOVA were utilized. A p-value of <0.05 was necessary to determine statistical significance. IBM SPSS Statistics for Windows, Version 22 (IBM Corp., Armonk, NY) was utilized to examine the information.

## Results

Prevalence of sexual dysfunction

The three groups examined in the study had a notable prevalence of SD, according to the research. In the group with affective disorders, 42% of participants displayed SD, while in the group with schizophrenia, the prevalence was notably higher at 64%. The control group, made up of individuals without psychiatric disorders, had a rate of 18%. These results suggest that SD is much more prevalent in psychiatric patients compared to the general population (Table 1).

**Table 1:** Prevalence of SD SD: sexual dysfunction

Group n (50)	Prevalence of SD				
	Without SD	SD	Total	Male	Female
Affective disorder	29	21	42%	36%	06%
Schizophrenia	18	32	64%	60%	04%
Control	41	09	18%	18%	00

In the group with affective disorders, the highest prevalence of SD was seen in specific areas: erection/vaginal lubrication (42%), arousal (42%), and difficulty achieving orgasm (42%). Likewise, the schizophrenia group had the highest prevalence in the areas of erection/vaginal lubrication (64%), arousal (60%), and desire/drive (60%). These findings by domain emphasize the diverse effects of psychiatric medications on various aspects of sexual function.

Socio-demographic correlates

Factors such as age, religion, education level, and socio-economic status were examined to determine their relationship with SD. In the group with affective disorders, age was significantly associated with SD (<0xCD><0x82>=14.613, df=3, p=0.002). Older participants reported higher rates of SD, possibly due to age-related changes and the effects of long-term medication use. Religion also showed a significant correlation with SD (<0xCD><0x82>=10.134, df=2, p=0.006), with differences noted among different religious groups. Education level and socio-economic status were also linked to SD in this group. 

Conversely, in the group with schizophrenia, no significant relationships were found between these sociodemographic factors and SD. This lack of association suggests that factors unique to the diagnosis of schizophrenia or its treatment may have a stronger impact on SD than sociodemographic variables. The severity of the illness and the use of multiple medications may be more influential in determining the incidence of SD in this group.

An analysis was performed to determine the association between SD and socio-demographic factors. In the group with affective disorder, there was a significant correlation (<0xCD><0x82>=14.613, df=3, p=0.002) between age and SD. The higher SD rates reported by participants in older age groups may be due to age-related physiological changes and the cumulative effects of long-term medication use. Furthermore, a significant correlation was found between religion and SD (<0xC8><0x82>=10.134, df=2, p=0.006), with varying trends observed among the religious groups. A significant relationship was observed (<0xCD><0x82>=22.234, df=6, p=0.001) between education level and SD, with higher rates of SD reported by those with lower education levels. Socioeconomic status was found to be a significant factor as well (2 = 12.134, df = 3, p = 0.007), with participants from lower socioeconomic backgrounds exhibiting a higher prevalence of SD. Conversely, no significant relationships were observed between these sociodemographic characteristics and SD in the schizophrenia group. The absence of correlation may indicate that influences specific to the diagnosis or treatment of schizophrenia outweigh the effects of sociodemographic variables on SD. The severity of the condition and the usage of several medications might be more significant variables in establishing the prevalence of SD in this population.

Duration of psychotropic use

The amount of time spent taking psychotropic drugs was found to have a significant impact on SD. In the group with affective disorder, prolonged drug use was found to be significantly associated with higher severity of SD (2 = 43.409, df = 8, p = 0.0000) (Table 2). In the schizophrenia group, too, longer medication use was associated with higher SD severity (2 = 24.800, df = 4, p = 0.000) (Table 3). Spearman correlation analysis showed that the length of drug use and the severity of SD had a significant positive correlation in both groups (r = 0.887 (p = 0.000) for the affective disorder group and r = 0.634 (p = 0.000) for the schizophrenia group). These findings suggest that the longer patients take psychotropic medications, the higher the likelihood that they will experience severe SD. This illustrates how important it is to regularly check on patients and adjust medication schedules as necessary to minimize adverse effects on sexual health. Clinicians should consider how long patients have been taking their medications and explore strategies to reduce these side effects when evaluating patients for SD.

**Table 2:** Association between duration of psychotropic use and sexual dysfunction in affective disorder group PRSexDQ: Psychotropic-Related Sexual Dysfunction Questionnaire, df: degree of freedom

Duration of drug use in months	PRSexDQ scale			Statistical result		
	Mild (27)	Moderate (11)	Severe (12)	Chi-square	df	P-value
1 to 40	27	04	00	43.409	8	0.000
41 to 80	00	07	09			
81 to 120	00	00	00			
121 to 160	00	00	01			
161 to 200	00	00	01			
201 to 240	00	00	01			

**Table 3:** Association between duration of psychotropic use and sexual dysfunction in the schizophrenia group PRSexDQ: Psychotropic-Related Sexual Dysfunction Questionnaire, df: degree of freedom

Duration of drug use in months	PRSexDQ scale			Statistical result		
	Mild (14)	Moderate (29)	Severe (07)	Chi-square	df	P-value
1 to 40	14	18	00	24.800	4	0.000
41 to 80	00	07	02			
81 to 120	00	04	05			
121 to 160	00	00	00			
161 to 200	00	00	00			
201 to 240	00	00	00			

Quality of life

The overall QoL was significantly lower in people with SD across all groups based on the Q-LES-Q-SF measurements. When SD was present, there was a significant decline in the affective disorder group's quality of life scores (Spearman correlation, r = 0.419, p = 0.002). The control group (r = 0.494, p = 0.000) and the schizophrenia group (r = 0.497, p = 0.000) showed comparable patterns (Tables 4-6).

**Table 4:** Correlation of sexual dysfunction with quality of life in the affective disorder group ASEX: Arizona Sexual Experience Scale

	Quality of life (% max score)	
Sexual dysfunction on ASEX	r value	0.419
	P-value	0.002

**Table 5:** Correlation of sexual dysfunction with quality of life in the schizophrenia group ASEX: Arizona Sexual Experience Scale

	Quality of life (% max score)	
Sexual dysfunction on ASEX	r value	0.497
	P value	0.000

**Table 6:** Correlation of sexual dysfunction with quality of life in the control group ASEX: Arizona Sexual Experience Scale

	Quality of life (% max score)	
Sexual dysfunction on ASEX	r value	0.494
	P-value	0.000

These findings highlight the substantial impact that SD has on the overall well-being and life satisfaction of psychiatric patients. Participants with SD expressed less satisfaction in many areas of life, including work, leisure activities, emotional stability, and physical health. The deleterious impacts of SD on QoL were more apparent in the schizophrenia group, where SD severity was higher. This suggests that treating SD is crucial for improving treatment outcomes, overall QoL, as well as sexual health in psychiatric patients. The results also highlight how critical it is to offer comprehensive care that addresses both mental and sexual health concerns. If SD is discussed during regular psychiatric care, it may be helpful to identify SD early and provide the right interventions. By increasing patient satisfaction and treatment compliance, this approach has the potential to improve mental health outcomes in the long run. 

## Discussion

Results from previous research support the high rate of SD among patients with mental illnesses on long-term psychotropic medication. The affective disorder group demonstrated a significant rate of SD, particularly in domains related to erection/vaginal lubrication, arousal, and orgasm [9,10], which indicates how pervasive these issues are in this population. The even higher prevalence observed in the schizophrenia group underscores the complex challenges these patients face, especially in light of the severe impairment of sexual function and overall QoL caused by the side effects of these medications [11,12]. The significant associations found between age, religion, education, and socioeconomic status and SD in the affective disorder group suggest that these factors may have a significant influence on the manifestation of SD. Our outcomes are consistent with earlier investigations that have demonstrated the influence of sociodemographic variables on the frequency as well as severity of SD [13,14]. Remarkably, no such associations were discovered in the schizophrenia group, suggesting that other factors, possibly related to the severity of the disorder or the specific medications taken, may have had a greater influence than the sociodemographic factors. 

Longer medication use has been associated with a higher severity of SD, indicating that the length of time spent using psychotropic medications is an important consideration. This research highlights how important it is to closely monitor long-term medication regimens in order to minimize harmful side effects associated with sexual activity [15,16]. The length of drug use and the severity of SD were found to have a significant positive correlation in both the affective disorder and schizophrenia groups [17]. This suggests that treatment plans may need to be adjusted to maintain sexual health and improve overall patient well-being. Because SD has a significant detrimental influence on QoL, doctors need to address these issues early on. Patients with SD expressed lower levels of satisfaction in many areas of their lives, which may exacerbate mental health problems and hinder their ability to heal. Remarkable treatment compliance and patient QoL can only be improved by effective management strategies like medication modifications and psychoeducation [18,19]. Psychiatric patients with SD require a multimodal approach that considers pharmaceutical and non-pharmacological interventions. Clinicians should investigate alternative treatments that lower the risk of SD in addition to talking with patients about potential medication side effects. Additionally important in controlling expectations and enhancing communication can be psychoeducation for patients and their partners. 

The role of non-pharmacological therapies, such as cognitive-behavioral therapy (CBT), in treating SD requires further investigation. CBT can help patients with anxiety, depression, and relationship issues by addressing the psychological aspects of SD. Furthermore, because they have been shown to improve sexual function, lifestyle modifications like regular exercise and eating a healthy diet should be encouraged as part of an all-encompassing treatment plan. Future studies should focus on larger and more diverse populations to gain a better understanding of the prevalence and effects of SD in different psychiatric groups. Longitudinal studies have the potential to shed light on the long-term consequences of psychotropic drugs on sexual health and may also suggest practical management and preventative strategies. Research comparing how different drugs and dosages affect sexual function may also be able to assist medical professionals in selecting the most appropriate treatment plans for individual patient cases. More targeted interventions have been required to decrease the adverse effects of psychiatric medications on sexual function. Drugs may be adjusted, dosages reduced, or substances intended to offset sexually transmitted infections added. These are a few instances of pharmacological tactics. The development of psychotropics that are less likely to result in SD could also be a significant advancement for the field. 

Educating medical professionals about the prevalence and implications of social distancing in mentally ill patients is also necessary. Sensitive and practical methods for addressing issues related to sexual health should be included in training programs. By expanding their knowledge and communication abilities, healthcare professionals can assist patients in managing SD more effectively as well as in enhancing their overall QoL. Furthermore, when it comes to information and support, patient advocacy and support groups are crucial resources for individuals with SD. These online communities can give patients a place to share their stories, get advice from other participants, and obtain resources to help them cope with their condition. Encouraging patients to join these groups can help them develop better-coping strategies and lessen the stigma associated with SD.

There are limitations, such as the small sample size and single-center study. We didn't include particular medications. As each drug has a particular effect on the body, this is a major drawback. Follow-up will be required for an extended period.

## Conclusions

According to this study, sexual dysfunction (SD) is prevalent among psychiatric patients receiving long-term medical care and has significant adverse effects on their quality of life (QoL). The findings highlight the need for more effective strategies for treating these side effects, which may enhance treatment compliance and general health. Future research should focus on large-scale studies, comparison of individual medications, and exploration of interventions to manage SD in psychiatric populations. To enhance their QoL and guarantee better treatment outcomes, psychiatric patients need to have their SD addressed. Psychiatrists can treat SD and assist patients in achieving the highest level of psychological and sexual well-being by employing holistic approaches that combine medical and non-medical treatments.
